# Meningoencephalomyelitis of Unknown Origin in Cats: A Case Series Describing Clinical and Pathological Findings

**DOI:** 10.3389/fvets.2020.00291

**Published:** 2020-05-22

**Authors:** Jasmin Nessler, Peter Wohlsein, Johannes Junginger, Florian Hansmann, Johannes Erath, Franz Söbbeler, Peter Dziallas, Andrea Tipold

**Affiliations:** ^1^Department of Small Animal Medicine and Surgery, University of Veterinary Medicine Foundation, Hanover, Germany; ^2^Department of Pathology, University of Veterinary Medicine Foundation, Hanover, Germany

**Keywords:** non-infectious, feline, lympho-histiocytic, MRI, necropsy, encephalitis, MUO

## Abstract

Meningoencephalomyelitis of unknown origin (MUO) is an umbrella term describing inflammatory changes of the central nervous system (CNS) with suspected non-infectious etiology. Diagnosis of MUO mostly remains presumed in a clinical setting. Histopathological and immunohistochemical examination of CNS tissue represent additional tools for detection of inflammation and the exclusion of specific infectious agents. While MUO is well-described in canine patients, only little is known about MUO in cats. Previous reports of feline MUO involve either clinical findings or histopathological examination but not both. The present case series is the first report describing both clinical and histopathological findings of feline MUO: Four cats (age: 1.7–17.8 years) showed acute to chronic progressive neurological signs of encephalopathy or myelopathy. Three cats had extraneural signs (hyperthermia, weight loss, hyporexia, leukocytosis). Magnetic resonance imaging (MRI) showed multifocal intraparenchymal lesions in forebrain, brainstem or spinal cord with homogenous contrast enhancement (2/2). Cerebrospinal fluid (CSF) examination was normal or displayed albuminocytologic dissociation. Histopathology revealed a multifocal, lympho-histiocytic meningoencephalitis in three cases and a lympho-histiocytic myelitis in one case. Immunohistochemistry for feline parvovirus, feline coronavirus, feline herpesvirus, tick borne encephalitis virus, Borna disease virus, morbillivirus, rabies virus, suid herpesvirus-1, and *Toxoplasma gondii* were negative in all cases.

**One Sentence Summary:**

This case series is the first one reporting both clinical and histopathological findings in cats with MUO. Feline MUO incorporates heterogeneous subtypes of sterile CNS inflammation.

## Introduction

Meningoencephalomyelitis of unknown origin (MUO) is an umbrella term describing inflammatory changes of the central nervous system (CNS) with suspected non-infectious etiology (1). However, the definitive diagnosis of a non-infectious meningoencephalomyelitis can only be made via histopathological examination and exclusion of known infectious agents using immunohistochemistry and/or molecular methods (1). In dogs, a broad range of different MUO are well-described and classified as for example granulomatous meningoencephalitis, necrotizing meningoencephalitis, or eosinophilic meningoencephalitis (1–5). In cats, encephalitis is frequently caused by infectious organisms (6, 7). Reports of MUO in cats are rare and focus on either clinical (8) or pathological aspects (9) of the disease and a classification system for feline MUO is missing. In addition, cases of feline meningoencephalitides are described, in which the authors suggest an infectious agent, but the agent itself was not detected (6, 7, 10).

This is the first case series, which describes both clinical and histopathological findings of cats with inflammatory changes in the CNS suggestive of MUO.

## Materials and Methods

A retrospective single-centered database search was performed to identify feline patients, which underwent diagnostic work up including cerebrospinal fluid (CSF) examination and/or magnetic resonance imaging (MRI) due to neurological signs and in which necropsy findings were suggestive of MUO presented from 2012 and 2019 in the Department of Small Animal Medicine and Surgery. In this period a total number of 10,954 cats were presented to of which 207 cats had either a CSF and/or MRI examination. Of those 207 animals in 38 cats a highly likely diagnosis of encephalitis of various origin was made because of CSF and MRI findings. In 11 of these cats MUO was presumed (Table 1). In four cats histopathological changes were suggestive of MUO. These cats were clinically investigated by at least one Resident or Diplomate of the European College of Veterinary Neurology (ECVN) and one Diplomate of the European College of Veterinary Anesthesia and Analgesia (ECVAA). All examinations were performed with informed written owners' consent according to the guidelines of the university. MRI examination (3.0 T MRI scanner Achieva, Philips Medical Systems, Best, The Netherlands; MRI sequences and planes presented in Table 2) and CSF collection were performed in general anesthesia. Necropsies, histopathology and immunohistochemistry were performed at the Department of Pathology and all cases were investigated by a Diplomate of the European College of Veterinary Pathologists (ECVP).

**Table 1:** Cats with presumed and confirmed diagnosis of encephalitis (2012–2019): comparison between cats with necropsy confirmed MUO, clinically suspected MUO, other encephalitides and clinic population.

	**Gender**	**Breed**	**Age Median (min-max)**	**Extracranial signs *n* = abnormal/all (cinical signs)**	**Blood examination leucocytes: median (min–max); *n* = abnormal/all other abnormalities**	**CSF Median (min-max); *n* = abnormal/all**	**MRI *n* = abnormal/all (MRI signs)**	**Treatment/outcome**	**Necropsy confirmed**
MUO, confirmed with necropsy (cases 1–4) *n* = 4	mn; *n* = 2 (50%) f; *n* = 1 (25%) fn; *n* = 1 (25%)	DSH; *n* = 3 (75%) Burmese; *n* = 1 (25%)	7 (1–17)	*n* = 2/4 (body temp 39.0–39.9°C *n* = 1, tachycardia *n* = 1, hyporexia/weight loss *n* = 1)	Leukocytes: 14.75 ^*^10∧3 cells/μl (6.1-24.9); *n* = 2/4	TP: 45.67 mg/dl (17–72); *n* = 2/3	*n* = 4/4 (multifocal *n* = 3/3; intraaxial *n* = 3/3; brain *n* = 2/3, spinl cord *n* = 1/3; contrast *n* = 2/2)	AED *n* = 1; anti-inflammatory *n* = 2; euthanasia after 1 week to 3 months *n* = 2; euthanasia *n* = 2	*n* = 4
					Hyperglycemia *n* = 1/4	Cells: 3 cells/3 μl (0–5); *n* = 0/3			
MUO, clinically suspected *n* = 7	m; *n* = 1 (14.3%) mn; *n* = 4 (57.1%) fn; *n* = 2 (28.6%)	DSH; *n* = 6 (85.7%) DLH; *n* = 1 (14.3%)	7.04 (0.3–15)	*n* = 1/7 (cardiac arrhythmia)	Leukocytes: 8.9 (6.1–12.6); *n* = 2/7	TP: 57.57 mg/dl (6–187); *n* = 5/7	*n* = 6/7 (multifocal *n* = 2/6; focal 4/6; intraaxial *n* = 6/6; brain *n* = 4/6, spinl cord *n* = 3/6; contrast *n* = 6/6)	AED *n* = 3; anti-inflammatory *n* = 5; euthanasia after 1 month *n* = 1, follow up 1.5–7 months *n* = 3; unknown *n* = 3	*n* = 0
					*n* = 5/7 (hyperproteinemia *n* = 2, increased liver enzyme activity *n* = 1; thrombocytopenia *n* = 1; increased Na^+^ *n* = 1)	Cells: 87.43 cells/3 μl (0–386); *n* = 2/7 Neutrophilic = 1, lymphocytic = 1			
Limbic encephalitis *n* = 6	m; *n* = 1 (16,7%) f; *n* = 2 (33.3%) fn; *n* = 3 (50%)	DSH; *n* = 5 (83.3%) MC; *n* = 1 (16.6%)	4.17 (2)	*n* = 1/6 (body temp 39.0–39.9°C *n* = 1)	Leukocytes: 11.76^*^10∧3 cells/μl (7.6–19.5); *n* = 2/5	TP: 18.6 mg/dl (14–24); *n* = 0/5	*n* = 6/6 (bilateral symmetric intraaxial *n* = 6/6; hippocampus *n* = 6/6; piriform lobe *n* = 2/6; contrast *n* = 3/6)	AED *n* = 3; follow up 3 weeks to 3 months; euthanasia *n* = 2	*n* = 1
					*n* = 2/5 (increased liver enzyme activity *n* = 2)	Cells: 3.83 cells/3 μl (1–9); *n* = 0/6			
FIP *n* = 9	m; *n* = 2 (22.2%) mn; *n* = 4 (44.4%) fn; *n* = 3 (33.3%)	DSH; *n* = 5 (55.5%) MC; *n* = 3 (33.3%) NF; *n* = 1 (11.1%)	2.5 (0.5–11)	*n* = 6/9 (body temp >40°C *n* = 2; body temp < 37°C *n* = 2; hyporexia/weight loss *n* = 3, dyspnoea *n* = 1)	Leukocytes: 14.85^*^10∧3 cells/μl (2.3–27.3); *n* = 4/8	TP: 1,129 mg/dl (745–2,200; *n* = 4/4), pandy pos *n* = 4/4	*n* = 7/7 (diffus thickening and contrast of epithelial lining and meninges *n* = 5/7; hydrocephalus *n* = 1/7; intramedullary spinal cord with contrast *n* = 2/7)	Euthanasia *n* = 8; unknown *n* = 1	*n* = 4
					*n* = 5/8 (hyperproteinemia *n* = 3; hypalbuminemia *n* = 2; increased liver enzyme activity *n* = 1; anemia *n* = 1)	Cells: 677.67 cells/3 μl (260–2,160); *n* = 6/6 Neutrophilic = 3, lymphocytic = 3			
Encephalitis secondary to otitis interna *n* = 7	m; *n* = 1 (14.3%) mn; *n* = 3 (42.9%) f; *n* = 1 (14.3%)fn; *n* = 2 (28.6%)	DSH; *n* = 2 (28.6%) MC; *n* = 3 (42.9%) Persian; *n* = 1 (14.3%) Siamese; *n* = 1 (14.3%)	4.88 (0.3–12)	*n* = 2/7 (vomitus *n* = 2)	Leukocytes: 12.5^*^10∧3 cells/μl (4.4–24.5); *n* = 3/7	TP: 374 mg/dl (0-1714); *n* = 3/5	*n* = 7/7 (unilateral middle ear effusion *n* = 6/7; bilateral middle ear effusion *n* = 1/7; focal intraaxial brain *n* = 2/7; focal extraaxial brain *n* = 2/7; contrast *n* = 7/7)	AB *n* = 6; antifungal *n* = 1; AED *n* = 1; follow up 1 week to 5 years *n* = 4; euthanasia *n* = 1; unknown *n* = 2	*n* = 1
					*n* = 2/7 (hyperproteinemia *n* = 2)	Cells: 1,593 cells/3 μl (0–7,936); *n* = 2/5 Mixed = 1, neutrophilic = 1			
Other bacterial encephalitis *n* = 2	m; *n* = 1 (50%) mn; *n* = 1 (50%)	DSH; *n* = 1 (50%) MC; *n* = 1 (50%)	5 (2–8)	*n* = 2/2 (body temp >40°C *n* = 2; dyspnoea *n* = 1)	Leukocytes:13.45^*^10∧3 cells/μl (15.5–14.5); *n* = 2/2	TP: 20.9 mg/dl *n* = 1	*n* = 2/2 (extraaxial mass *n* = 1; multifocal intraaxial *n* = 1; fracture parietal bone *n* = 1; contrast *n* = 2)	AB *n* = 2; follow up 2 years *n* = 1; euthanasia *n* = 1	*n* = 0
					*n* = 2/2 (hypalbuminemia *n* = 1; increased Na^+^ *n* = 1)	Cells: 1,197 cells/3 μl (94–2,300); *n* = 2/2 Neutrophilic = 2			
Toxoplasmosis *n* = 1	f; *n* = 1 (100%)	DSH; *n* = 1 (100%)	1	*n* = 1/1 (vomitus, body temp >40°C, sneezing)	Leukocytes: 25.1^*^10∧3 cells; *n* = 1/1	TP: 17 mg/dl; *n* = 0/1	np	AB; follow up 6 months	*n* = 0
					*n* = 1/1 (hypalbuminemia)	Cells: 20 cells/3 μl; *n* = 1/1			
Presumed inflammatory brain lesion^*^ *n* = 2	fn; *n* = 2 (100%)	DSH; *n* = 1 (50%) BSH; *n* = 1 (50%)	6 (4–8)	Abnormal *n* = 1/2 (body temp 39.0–39.9°C *n* = 1)	Leukocytes: 13.15^*^10∧3 cells/μl (8.5–17.8); *n* = 1	TP: 49.5 mg/dl (31-68); n=2/2	*n* = 2/2 (multifocal *n* = 1; focal *n* = 1; contrast *n* = 1)	Dead after 1 week *n* = 1, euthanasia *n* = 1	*n* = 0
					*n* = 2/2 (hyperproteinemia *n* = 1; thrombocytopenia *n* = 1, increased liver enzyme activity *n* = 1)	Cells: 44.5 cells/3 μl (5–84); *n* = 1/2 Lymphocytic = 1			
All cats in the clinic 2012–2019 *n* = 10,954	m 9.2% mn 46.8% f 10.1% fn 33.9%	DSH 69.8% BSH 6.7% MC 6.0% Persian 3.7% Siamese 1.5% NF 1.4% DLH 0.38% Burmese 0.15% Others 10.4%							

*MUO, meningoencephalomyelitis of unknown origin; FIP, feline infectious peritonitis; m, male; mn, male neutered; f, female; fn, female neutered; min, minimum; max, maximum; DSH, Domestic Shorthair Cat; DLH, Domestic Longhair Cat; MC, Maine Coon Cat; NF, Norwegian Forrest Cat; BSH, British Shorthair Cat; body temp, rectal body temperature; CSF, cerebrospinal fluid; TP, total proteine; neutrophilic, neutrophilic pleocytosis, lymphocytic, lymphocytic pleocytosis; mixed, mixed cell pleocytosis; pandy pos, positive Pandy's reaction*.

* CSF and MRI suspicious of inflammatory CNS disease, but owner refused further investigation; leukocytes, reference range 6–11^*^10∧3 cells/μl; MRI, magnetic resonance imaging findings; contrast, contrast enhancement; np, not performed; euthanasia, euthanasia after diagnosis; AED, antiepileptic drug (levetiracetam, phenobarbital); AB, antibiotic drug; antifungal, itaconazol; anti-inflammatory, cytarabin and/or prednisolone; follow up, last known time alive; unknown, lost to follow up.

**Table 2:** Magnetic resonance imaging (MRI) sequences and planes used for each cat.

	Cat 2^*^	**Cat 3**	**Cat 4**
Scanned region	Brain	Brain	Spinal cord, brain
T2w	trans, sag, dor	trans, sag, dor	trans (T7-L1), sag (olfactory bulb to Cd5)
T1w pre-contrast	n.d.	trans, sag, dor	n.d.
T1w post-contrast^a^	n.d.	trans, sag, dor	trans (T11-13), sag (C5-L7)
GRASE	trans	trans	trans (T9-L1)
FLAIR	n.d.	trans	n.d.
DWI/ADC	n.d.	trans	n.d.
Fast spin echo	n.d.	n.d.	trans (T9-L1)

*T2w, T2 weighted; T1w, T1 weighted; DWI/ADC, diffusion weighted imaging/apparent diffusion coefficient; GRASE, Gradient and Spin Echo; FLAIR, Fluid-attenuated inversion recovery (=Long Tau Inversion Recovery); trans, transversal plane; sag, sagital plane; dor, dorsal plane; T, thoracic spinal segment; L, lumbar spinal segment; C, cervical spinal segment; Cd, caudal spinal segment*.

* *Scans were performed post-mortem; n.d., not performed*.

a *Contrast medium (gadoteric acid 1 mmol/kg, megluminum 195 mg/kg, Dotarem®, Guerbet AG, Zürich, Swiss)*.

## Cases

### Cat No. 1

A 25-months-old domestic shorthair cat (DSH), male-neutered, was presented due to 1 week history of hyporexia and hyperthermia. One year before initial presentation, the cat was imported from China without any clinical abnormalities. Vaccination and deworming history was not traceable.

In the general examination, the rectal temperature was 39.2°C, tachycardia (200 bpm) was present, and the cat was constantly vocalizing. In the neurological examination, he was obtunded and disorientated and showed compulsive pacing. Handling elicited a generalized increased muscle tone and whole body tremor. A diffuse intracranial lesion was suspected. Metabolic/toxic and inflammatory etiologies were suspected differential diagnoses.

Blood count, liver enzymes, blood glucose, urea, creatinine, protein, albumin, preprandial ammonia, and plasma electrolyte concentrations were within the reference range. Plasma thiamine concentration were below the reference level [32 μg/l, reference > 50 μg/l (11)]. With the exception of hyporexia no reason for thiamin deficiency could be found in the history.

Supplementation of thiamine [20 mg/kg subcutaneously (s. c.) twice daily (BID)] and symptomatic therapy as hand feeding and fluid corrections if needed did not improve clinical signs within 1 week. The owner refused MRI examination due to financial constraints. Examination of suboccipitally collected CSF was without changes (protein 17.25 mg/dl, glucose 63 mg/dl, 1 leukocyte/3 μl, 6 erythrocytes/3 μl). Because of worsening of clinical signs and unclear prognosis owner decided for humane euthanasia.

**Figure 1:**
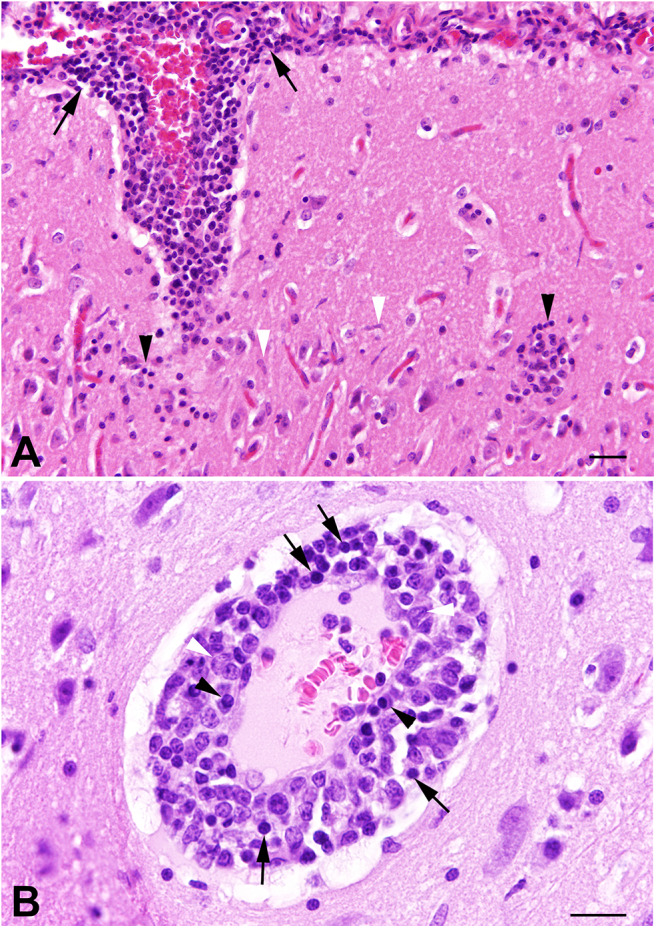
Cat no. 1. **(A,B)** Histopathology. **(A)** Cerebral cortex, the leptomeninx (arrows) and neuroparenchyma (black arrowheads) is severely infiltrated with mononuclear inflammatory cells associated with rod-shaped microglial cells (white arrows) in the adjacent neuroparenchyma. HE, bar = 50 μm. **(B)** Cerebral cortex showing severe vasculitis with mural and perivascular infiltration of lymphocytes (arrows), macrophages (white arrowheads), and plasma cells (black arrowheads). HE, bar = 60 μm.

Histology revealed a multifocal moderate lympho-histiocytic and plasmacytic meningoencephalomyelitis and vasculitis (Figure 1). Occasionally, neuronal necroses were found. Particularly adjacent to affected blood vessels a moderate microgliosis with rod-shaped microglial cells was present in the neuroparenchyma (Figure 1). In the spinal cord, moderate numbers of swollen axons within dilated myelin sheaths were detected in dorso-lateral, lateral and ventral funiculi.

The lung of the cat showed a granulomatous inflammation with necrotizing vasculitis.

Immunohistology for feline parvovirus, feline coronavirus, feline herpesvirus, tick borne encephalitis virus, Borna disease virus, morbillivirus and rabies virus was negative in CNS parenchyma.

### Cat No. 2

A female neutered DSH, 17 years and 10 months of age, was presented with acute progressive asymmetric neurological deficits. The cat had regular outdoor access, was regularly vaccinated and dewormed and had no history of any other disease. Trauma could not be excluded.

General examination was normal. In the neurological examination, obtundation and ambulatory tetraparesis with proprioceptive ataxia and ventroflexion of the head were obvious. Both pupils were poorly responsive to light with an anisocoria (right pupil mydriatic). The neuroanatomical localization was midbrain and brainstem with a right sided accentuation. Most important differential diagnoses were inflammatory, neoplastic or traumatic lesions.

Blood examination revealed a normal blood count, hyperglycemia (223 mg/dl, reference <129 mg/dl) and mild hypokalemia (2.91 mg/dl, reference range 3–4.8 mg/dl) with normal urea, creatinine and liver enzymes. Before further examinations were performed, the cat suffered from a peracute respiratory arrest and lost consciousness. Immediate intubation and mechanical ventilation was performed. Normal sinus rhythm with a heart rate in reference range was seen in electrocardiographic examinations. Radiographs of the thorax showed a moderate alveolar lung pattern of the dorsal aspects of the left and right caudal lung lobes not responding to furosemide bolus injection (2 mg/kg i.v.). A neurogenic lung edema was suspected. The cat was comatose with absent vestibulo-ocular and palpebral reflexes. Both pupils were not responsive and remained in moderate mydriasis. Brainstem auditory evoked response [modified according to Plonek et al. (12)] showed normal wave I, III and V latencies on both sides. Electroencephalography [modified according to Brauer et al. (13)] displayed sporadic spikes, a constant underlying beta rhythm and occasionally muscle artifacts caused by swallowing. The cat did not regain consciousness or spontaneous breathing within 12 h of mechanical hypocapnic ventilation and after mannitol bolus infusion (1 g/kg i. v.). Due to poor prognosis, the owner decided for humane euthanasia.

**Figure 2:**
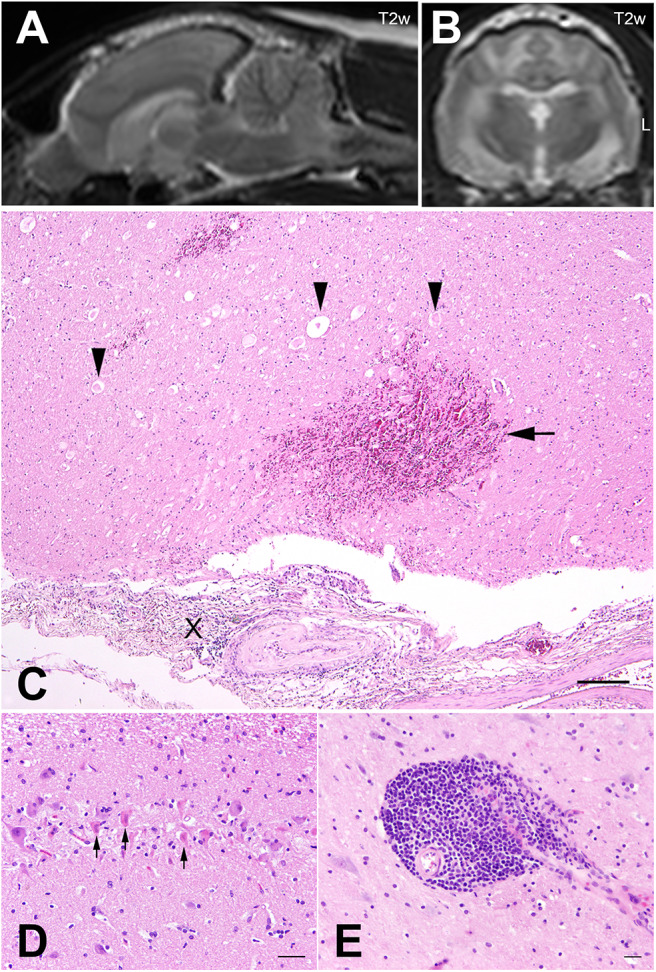
Cat no. 2. **(A,B)** Magnetic resonance imaging (MRI): Midsagittal **(A)** and transversal section **(B)** of the brain of cat 2 at the level of the interthalamic adhesion in T2 weighted (T2w) sequences. Note the intramedullary hyperintense lesions of the brainstem and the subcortical white matter as well as the mass lesion with signal void in the myelencephalon. Left side is marked with L. **(C–E)** Histopathology. **(C)** Pons, focally severe meningitis (X) with severe hemorrhages in the adjacent neuroparenchyma (arrow) and vacuolization of the white matter as well as spheroids (arrowheads), HE, bar = 100 μm. **(D)** Hippocampus, severe neuronal necrosis (arrows) and loss. HE, bar = 50 μm. **(E)** Cerebral cortex with focally severe, perivascular, lympho-histiocytic encephalitis. HE, bar = 60 μm.

Post-mortem MRI revealed multifocal bilateral asymmetric intraaxial lesions without mass effect in the subcortical white matter of both telencephalic hemispheres, left olfactory bulb and the brainstem. The lesions were hyperintense to normal white matter in T2 weighted (T2w) and gradient and spin echo (GRASE) sequences, partly well-demarcated and partly ill-demarcated. Additionally, a well-demarcated intraaxial lesion in the myelencephalon reaching into the cranial cervical spinal cord displaying mild mass effect with signal void in T2w and GRASE was visible (Figure 2), which was surrounded by a hyperintense area additionally involving the ventral area of the cerebellar vermis.

Histopathology of the brain revealed acute focal extensive segmental neuronal necrosis of deep cortical neurons and neurons of the hippocampal CA2 region (Figure 2) associated with mild edema of the adjacent neuropil and hypertrophy of capillary endothelial cells. At the level of the pons a severe, focal, predominantly lymphocytic perivascular meningitis with extensive meningeal hemorrhage extending into the adjacent subpial neuroparenchyma and acute degeneration and necrosis of the white matter (liquefactive necrosis) associated with myelin sheath dilatation, axonal swellings and infiltration of few neutrophils and gitter cells was detected (Figure 2). An adjacent nucleus of the pontine gray matter showed acute neuronal necrosis. In addition, multifocal acute perivascular and mural hemorrhages were present particularly in the brain stem. Additional lesions consisted of multifocal, mild to severe, perivascular lympho-histiocytic meningoencephalitis (Figure 2). Immunohistochemistry targeting feline herpesvirus, feline leukemia virus, Borna disease virus, suid herpes virus 1, tick borne encephalitis virus, rabies virus and *Toxoplasma gondii* in CNS parenchyma were negative.

Extraneural lesions included mild multifocal chronic interstitial nephritis, nodular hyperplasia of thyroidal and adrenal glands interpreted as age-related changes without significant clinical relevance, and alveolar oedema and emphysema in the lung, which was most likely an agonal change but might also have been a neurogenic edema.

### Cat No. 3

A male-neutered DSH, 8 years and 5 months of age, was presented with paroxysmal episodes of abnormal behavior for 6 months. Episodes lasted for several minutes. During these episodes, the cat walked aimlessly or in circles to the right in a crouched position. Reduced consciousness and mydriatic pupils were obvious. Between the episodes, the owner did not recognize any neurological abnormalities. The cat significantly lost weight (~1.5 kg) despite normal appetite. Body condition score at presentation was 4–5 out of 9. In the history, regular outdoor access and regular vaccination and deworming were mentioned.

General examination was unremarkable. In the neurological examination, the cat seemed mildly obtunded but otherwise unremarkable. Reactive seizures or structural epilepsy were suspected.

Complete blood count, liver enzymes, blood glucose, urea, creatinine, protein, albumin, preprandial bile acids, plasma electrolyte, thiamine, cobalamin, and folic acid concentrations were within normal range.

**Figure 3:**
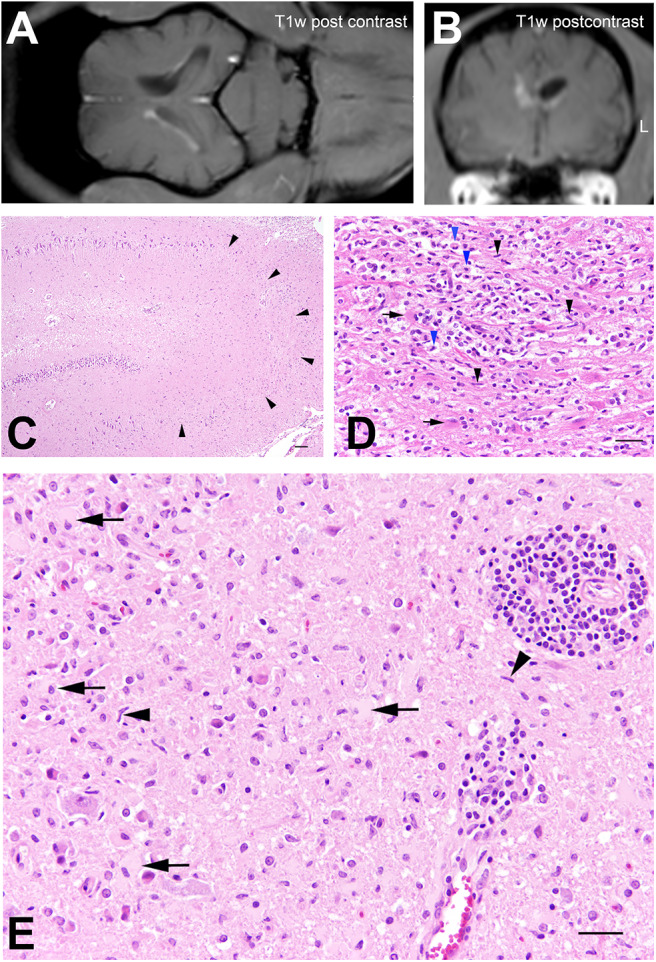
Cat no. 3. **(A,B)** Magnetic resonance imaging (MRI): Dorsal **(A)** and transversal section **(B)** of the brain at the level of the interthalamic adhesion in T1w sequences post-contrast application. Note the small rim of contrast enhancement in the lateral ependymal lining of the right lateral ventricle and the slightly decreased volume of the right lateral ventricle. Left side is marked with L. **(C–F)** Histopathology. **(C)** Hippocampus with severe neuronal loss in the CA2 and CA3 regions (arrowheads). HE, bar = 100 μm. **(D)** Periventricular white matter with severe microgliosis (black arrowheads), infiltration of gitter cells (blue arrowheads) and gemistocytes (arrows). HE, bar = 50 μm. **(E)** Brain stem, moderate lympho-histiocytic perivascular encephalitis with prominent microgliosis (arrowheads), astrocytosis and presence of gemistocytes (arrows). HE, bar = 60 μm.

MRI (Table 1) of the brain revealed only a small focal intraaxial lesion with mild mass effect in the ventro-lateral ependymal lining of the right lateral ventricle, isointense in T2w and T1w to normal white matter, hyperintense in fluid attenuated inversion recovery (FLAIR), and with marked homogenous contrast enhancement (Figure 3). Suboccipital CSF examination displayed normal cell count but slightly increased amount of granulocytes (5 white blood cells/3 μl, reference <15 white blood cells/3 μl, 72% lymphocytes, 11% neutrophils, 17% monocytes) and increased protein (48.53 mg/dl, reference <25 mg/dl). SNAP-Test for FIV and FeLV (Idexx, Ludwigsburg, Germany) in serum was negative, as well as antibody titers for *Toxoplasma gondii* (immunofluorescence antibody test) in serum (Laboklin, Bad Kissingen, Germany). Polymerase chain reaction (PCR) in CSF was negative for *Mycoplasma haemofilis, haemomintum*, and *turicensis*, Corona virus, and Borna virus (Laboklin, Bad Kissingen, Germany). MUO was suspected. Differential diagnosis included neoplasia like ependymoma or round cell tumor. No extracranial signs of lymphoma were found in abdominal sonography.

Therapy included anti-inflammatory treatment with prednisolone 1 mg/kg SID orally (p. o.) and anticonvulsive therapy with levetiracetam 30 mg/kg TID p. o. Several modifications of antiepileptic and anti-inflammatory medication did not lead to improvement (increased prednisolone up to 2.3 mg/kg BID; Imepitoin 11 mg/kg BID raised up to 22 mg/kg BID, Pexion®, Boehringer Ingelheim, Ingelheim am Rhein, Germany; add-on application of Phenobarbital 1 mg/kg BID, Luminaletten vet, Virbac, Carros, France; Diazepam 1 mg/kg rectally if necessary, Diazepam Desitin® rectal, Desitin Arzneimittel GmbH, Hamburg, Germany). MRI and CSF examinations were repeated after 3 months, but findings remained almost unchanged. The size of the lesion was only mildly increased compared to the first MRI and very mild unilateral middle ear effusion was visible. CSF protein concentration was 42.19 mg/dl, cells could not be detected in the examined sample. Due to increased seizure frequency and development of generalized tonic-clonic status epilepticus, the owner decided for humane euthanasia.

Histology revealed a multifocal mild to moderate, perivascular lympho-histiocytic meningoencephalitis particularly in the cerebral cortex and in the brainstem occasionally associated with prominent astro- and microgliosis (Figure 3). Within these areas numerous gemistocytic astrocytes were observed. Furthermore, small segments of the deep cerebral cortex displayed neuronal necrosis with mild vacuolisation of the neuropil. The white matter of the parietal cortex showed a dilatation of myelin sheaths, swollen axons and few myelinophages. Hippocampal CA2 and CA3 sectors showed extensive loss of neurons associated with mild astro- and microgliosis and the periventricular white matter of the parietal and temporal cerebrum was affected by severe partly nodular, partly diffuse microglial and astroglial proliferation with numerous gemistocytes and gitter cells (Figure 3). The diencephalon showed a diffuse, moderate astro- and microgliosis with numerous gemistocytes. In addition, a focal moderate perivascular lympho-histiocytic chorioiditis was present. Spinal cord sections showed no morphological alterations.

Immunohistochemistry targeting Borna disease virus, feline corona virus, suid herpesvirus I and *Toxoplasma gondii* was negative in CNS parenchyma.

Extraneural lesions consisted of severe granulomatous otitis media with cholesterol deposits.

### Cat No. 4

A 30-months-old entire female Burmese cat was presented with chronic progressive gait abnormalities of the pelvic limbs starting with a plantigrade stance. The cat had regular outdoor access. Vaccination and deworming history was not traceable.

General examination was unremarkable. Neurological examination revealed an ambulatory paraparesis with hypermetria and proprioceptive ataxia of the pelvic limbs. Spinal reflexes, cranial nerves and state of consciousness were normal. The cat showed no signs of pain on spinal palpation. A T3-L3 myelopathy was suspected. At this time point, the owner decided against further diagnostic work up. At the recheck appointment 4 weeks later the cat was non-ambulatory paraparetic with reduced muscle tone and withdrawal reflexes of the pelvic limbs. Anal and tail tone were also reduced. Cutaneous trunci reflex was completely absent on both sides. The cat still showed no signs of pain. The progression lead to the suspicion of a diffuse C8-S3 myelopathy.

Complete blood cell count revealed a neutrophilic leukocytosis [24.89 × 10^3^ cells/ml, reference range <12 × 10^3^ cells/ml of which 20.8 × 10^3^ cells/ml were neutrophilic granulocytes, 2.8 × 10^3^ cells/ml lymphocytes, 0.4 × 10^3^ cells/ml monocytes, and each 0.01 × 10^3^ cells/ml basophilic granulocytes and large unstained cells (LUC)]. Liver enzymes, blood glucose, urea, creatinine, protein, albumin, and plasma electrolyte concentration were within normal limits.

**Figure 4:**
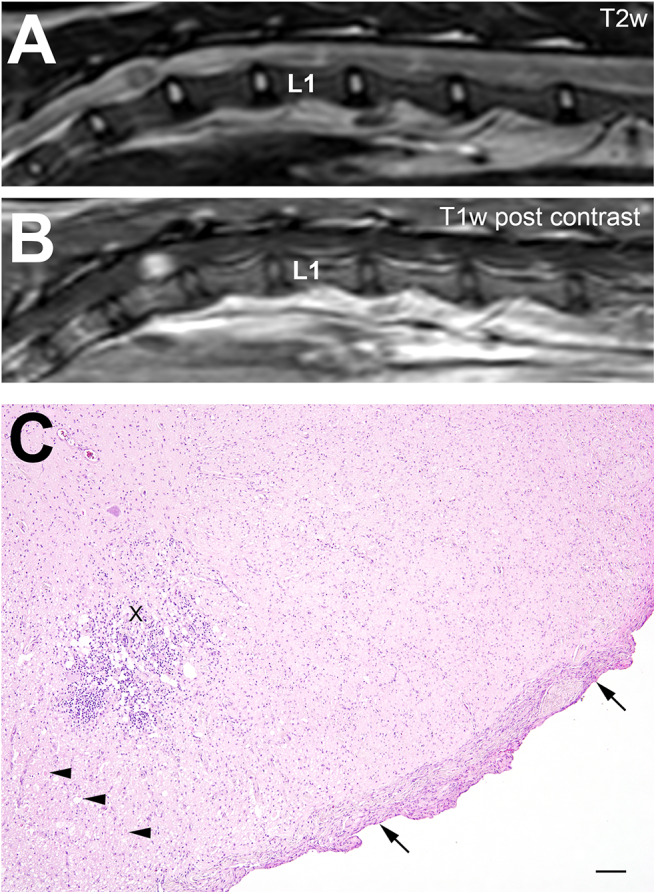
Cat no. 4. **(A,B)** Magnetic resonance imaging (MRI): Midsagittal section of the spinal cord at the thoracolumbar junction in T2w **(A)** and T1w post-contrast **(B)** sequences. First lumbar vertebra is marked with L1. Note the intramedullary hyperintense signal **(A)** from T11 to L1 and the focal contrast enhancement at the level of T12 **(B)**. **(C)** Histopathology: Thoracic spinal cord showing a focally extensive severe lympho-histiocytic inflammation (X) associated with dilatation of myelin sheaths and swollen axons (arrowheads). In addition, a severe meningeal fibrosis is present (arrows). HE, bar = 100 μm.

MRI examination showed a diffuse, intramedullary, poorly demarcated lesion reaching from T11 to L1 with mild mass effect. The lesion was heterogeneous hyper- and isointense to normal gray matter in T2w, GRASE and multiecho Fast Field Echo (mFFE) and isointense in T1w to normal white matter. The lesion was bilaterally asymmetric and involved white and gray matter. Only a small round well-demarcated intramedullary area at the level of T12 displayed marked homogeneous contrast enhancement (Figure 4). Examination of lumbar CSF showed albuminocytologic dissociation (no cells, protein 72.01 mg/dl, reference <40 mg/dl). Neoplasia or less likely myelitis was suspected. The owner declined biopsy and decided for humane euthanasia.

At the level of T11-13 a severe circumferential meningeal fibrosis including all layers extending into the spinal neuroparenchyma was found (Figure 4). Particularly around intraspinal blood vessels, a prominent increase of collagen fibers was present. A unilateral severe lympho-histiocytic myelitis of the ventral horn and the dorsal funiculus was observed. At the ventral horn, additional mild neuronal loss and associated degenerative changes of the adjacent white matter characterized by dilated myelin sheaths axonal losses and swellings were found. Cells showed no signs of malignancy.

Immunohistochemistry for Borna disease virus, feline coronavirus, suid herpesvirus 1, tick borne encephalitis virus and *Toxoplasma gondii* in CNS parenchyma was negative.

Considerable extraneural lesions included a moderate eosinophilic cystitis.

### Summary of the Cases

In four cats histopathological confirmed MUO was presented. Median age was 7 years, which is older than the median age of cats which presented to the clinic due to infectious encephalitis (1–4.88 years, Table 1). Cats with clinically suspected MUO had a similar median age (7.04 years, Table 1) as the presented ones with necropsy confirmed MUO. Breed distribution of the presented cases was comparable with general clinical population, while in cats with infectious encephalitis pedigree cats seemed to be more common (Table 1). In addition to acute to chronic neurological signs, systemic signs of illness or blood leukocytosis were present in 3/4 cats with confirmed MUO, in 3/7 with suspected MUO and in from 6/9 to 2/2 in infectious encephalitis (Table 1). MRI showed multifocal intraparenchymal lesions in the CNS with contrast enhancement in cats with MUO (Table 1), middle ear effusion with and without signs of ipsilateral meningeal contrast enhancement and intraaxial lesions in cats with encephalitis following otitis (Table 1), diffuse thickening and contrast enhancement of epithelial lining and meninges or intramedullary spinal cord lesions in cats with FIP (Table 1), or bilateral symmetrical lesions of the hippocampus with no to moderate contrast enhancement in cats with limbic encephalitis (Table 1). CSF changes seemed more subtle than in infectious encephalitis, with albuminocytologic dissociation being the most common finding (Table 1). Only cats with limbic encephalitis showed a normal CSF (Table 1). Histopathology revealed a multifocal, lympho-histiocytic inflammation in the CNS. Infectious agents were excluded via blood- and CSF examination as well as via IHC.

## Discussion

In the present case series different manifestations of histopathologically confirmed MUO in cats are described, differing in the cats' age, history, clinical and imaging findings and progression.

Median age of cats with confirmed MUO was 7 years as it was in cats with suspected MUO. In other studies cats with MUO were older [median 9.4 years (8)] or younger [median 5 years (7)], but the age range was similar (8). DSH were affected most often as they were in Negrin's study (8), which is comparable to the general hospital population. But cats with infectious encephalitis were pedigree cats in almost 50% of the cases, which confirms a study by Bradshaw et al. (6), where Burmese cats were overrepresented in cats with FIP. Two cats with MUO showed extraneural clinical findings as anorexia, weight loss, and increased body temperature. This seems less common and less severe than in most cases with infectious encephalitis, where up to 100% of cats per group showed extraneural clinical signs as pyrexia >40°C, hypothermia, vomitus, hyporexia and weight loss, dull hair coat and others. Negrin et al. (8) reported anorexia in 4/16 and pyrexia in 3/16 cats with MUO, while Singh et al. (7) showed extraneural signs in 4/5 cats with MUO. Leukocytosis occurred in 2/4 cats with confirmed MUO, which seems to be similar in all groups of encephalitis. But extraneural signs and leukocytosis seems to be more common in cats with MUO than in dogs (2). MRI (3/4 cats) showed very subtle to obvious multifocal bilateral asymmetric intraaxial lesions in forebrain, brainstem or spinal cord with homogenous contrast enhancement where this examination was performed (2/3). Negrin et al. (8) also reported multifocal lesions in MRI in brain and/or spinal cord with contrast enhancement in most cases (11/16). MRI findings matched the neuroanatomical localization of clinical signs as it mostly did in Negrin's study (8), but were not completely confirmed by histopathological findings, a phenomenon also known in canine MUO (1). Compared to infectious encephalitis, CSF changes are rather subtle and showed no changes or albuminocytologic dissociation. Necropsy findings were heterogeneous as well, although a perivascular lympho-histiocytic inflammation was common. Similar clinical and imaging were previously reported for feline MUO (8). But CSF pleocytosis was much more common in the report of Negrin et al. (8), presumably because this was one of the main inclusion criteria in this study. In canine MUO 12.5–22% of cases present without CSF pleocytosis and 3.2% with albumino-cytologic dissociation (1). Additionally, the outcome in the study from Negrin et al. was more favorable (8) due to the fact, that in the present case series only histopathologically confirmed cases with MUO were included. The heterogeneity of clinical signs and diagnostic findings makes the diagnosis of feline MUO difficult in a clinical setting. The diagnosis can only be presumed without a brain biopsy. Systemic involvement of blood vessels was seen in two cats histopathologically. This was not reported so far. Feline MUO seems to be a more systemic disease, since extraneural findings were detected during clinical examination and necropsy.

Other studies describing cases of feline MUO reported either only clinical signs (8) or only extensive post-mortem diagnostic workup (9). In studies about clinical and histopathological findings only marginal or no search for infectious agents was performed (6, 7, 10). In the current case series, a symbiosis of clinical signs and extensive histopathological workup could be achieved. Extensive diagnostic workup with search for specific infectious agents via immunohistochemistry was not able to disprove the hypothesis of feline MUO (5). However, such negative findings searching infectious agents do not exclude an infectious etiology since a presently unknown pathogen may be the causative agent of the disease. Furthermore, an infectious agent may have already been cleared at the time point of examination and the detected inflammation represents the sequela of the immunoreaction as shown, e.g., in human cases of fatal tick borne encephalitis virus infection (14). Nevertheless, it is currently suggested that MUO have a multifactorial etiology including an underlying genetic susceptibility and involving an additional unknown external trigger (5). In several dog breeds, as Pug dogs, Maltese, and Chihuahua, a genetic defect in DLA-II is known to increase the risk of developing MUO (3, 15). In cats, no such defect has been investigated so far. Future studies on feline cases suggestive of MUO should include additional molecular techniques, such as next-generation sequencing and discuss these results in the context of clinical and histopathological findings.

In general, feline MUO is less documented in literature than the canine counterpart (9). As feline MUO seems more heterogeneous, extensive diagnostic work up and conscientious search for infectious agents is essential to contribute to our knowledge about feline MUO.

## Data Availability Statement

All datasets generated for this study are included in the article/supplementary material.

## Author Contributions

JN examined cases and drafted the report. JE examined the cases. FS was responsible for critical care management of cases. PD performed the diagnostic imaging and described findings. JJ, FH, and PW performed the pathology and histopathology and described the findings. AT supervised the work and finalized the report.

## Conflict of Interest

The authors declare that the research was conducted in the absence of any commercial or financial relationships that could be construed as a potential conflict of interest.

## Abbreviations

BID: twice a day (lat. “*bis in die*”)
bpm: beats per minute
°C: degree celsius
CSF: cerebrospinal fluid
ECVAA: European College of Veterinary Anesthesia and Analgesia
ECVN: European College of Veterinary Neurology
ECVP: European College of Veterinary Pathologists
dl: deciliter
DSH: domestic shorthair cat
FeLV: feline leukemia virus
FIV: feline immunodeficiency virus
FLAIR: fluid attenuated inversion recovery
g: gram
GRASE: Gradient and Spin Echo
i. v.: intravenously
kg: kilogram
mFFE: Multiecho Fast Field Echo
mg: milligram
ml: milliliter
μg: microgram
l: liter
MRI: magnetic resonance imaging
PCR: polymerase chain reaction
p. o.: orally (lat. “per os”)
s. c.: subcutaneously
T1w: T1-weighted
T2w: T2-weighted
TID: three times a day (lat. “*ter in die*”).
